# Supplementary material for: Fleeing is believing: adaptive behavior under social threat as an inference process
Source: Front Neural Circuits. 2026 Jun 12;20:1812938. doi: 10.3389/fncir.2026.1812938 (PMC13303489; doi:10.3389/fncir.2026.1812938)
Supplement: Supplementary file 1 [file Data_sheet_1.pdf]

# Supplementary Material

## Fleeing is Believing: Adaptive behavior under social threat as an inference process

Hridai S Khurana[1,2], Valeria Mussetto[1], Leiron Ferrarese[1], Cornelius T Gross[1], Rory J Bufacchi[1*]

1. Epigenetics & Neurobiology Unit, EMBL Rome, European Molecular Biology Laboratory, Via Ramarini 32, 00015 Monterotondo (RM), Italy
2. Department of Biotechnology, Indian Institute of Technology Madras, Chennai 600036, India

* Corresponding author: rory.bufacchi@embl.it

# 1. Results

## 1.1. Parameter Recovery

Recovery was strong for *k_shelter* (r = 0.90) and *sensory slope* (r = 0.84), indicating that shelter occupancy and investigation patterns together reliably constrain these parameters. *ID threshold* showed moderate recovery (r = 0.64), sufficient for group-level comparisons but warranting caution at the level of individual subjects. *k_threat* (r = 0.42) and *bias_stay* (r = 0.37) were more poorly recovered, reflecting a structural degeneracy: strong shelter preference and strong threat aversion produce overlapping behavioral signatures under the current metric set, making these parameters difficult to separate [Supplementary Figure S1(A–C)]. Noise sensitivity analysis confirmed that *k_shelter* and *sensory slope* remain well-identified under moderate simulation stochasticity, while poor recovery of *k_threat* and *bias_stay* is independent of noise and therefore reflects a fundamental property of the metric space rather than simulation variance [Supplementary Figure S1(B)]. The parameters most directly relevant to the paper's principal findings - shelter-seeking drive and sensory acuity - are therefore reliably identified. The poorly-recovered parameters may contribute to the model's ability to fit diverse behavioral phenotypes but should not be interpreted in isolation from aggregate metrics alone.

*Supplementary Figure S1*

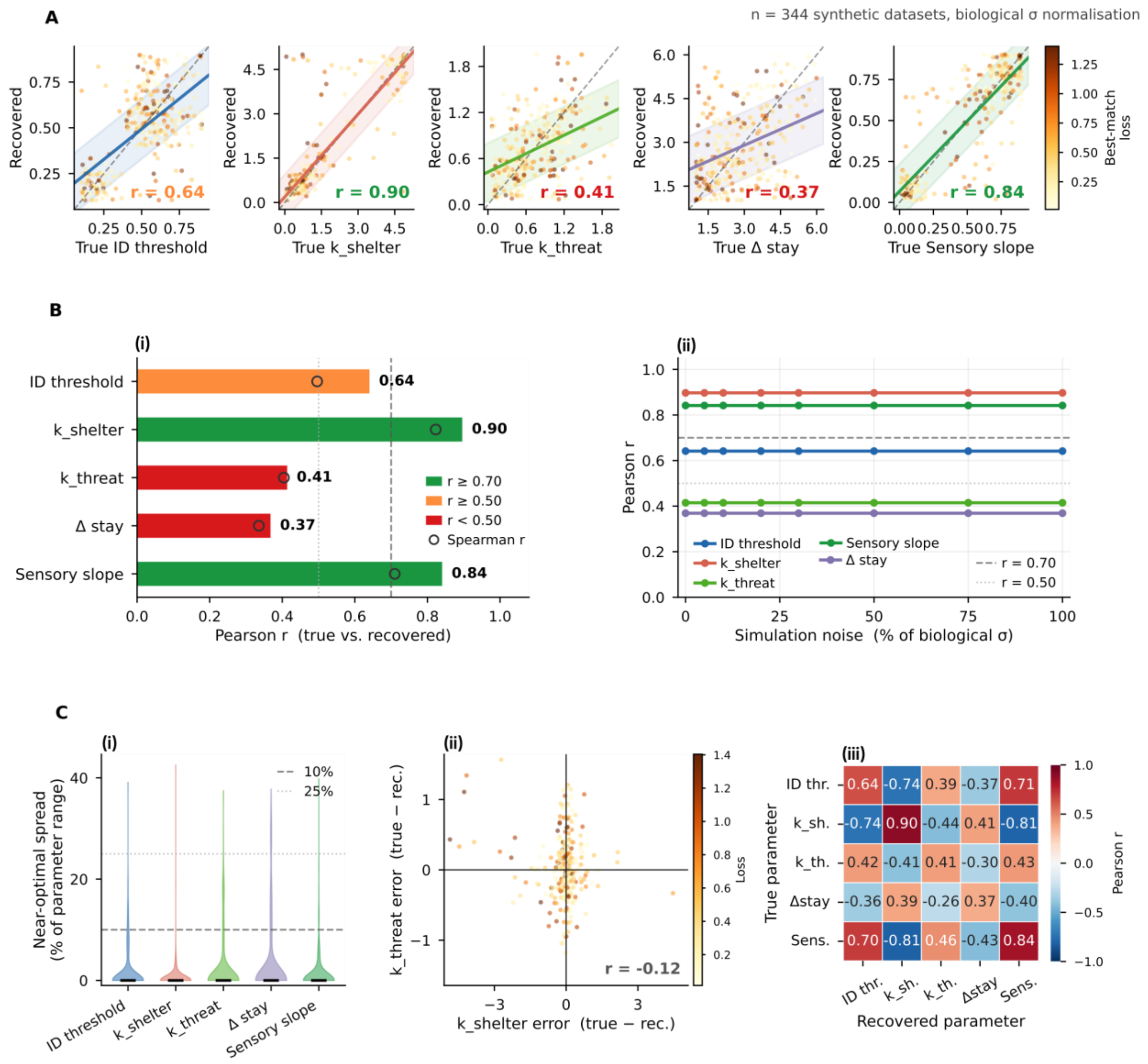


Supplementary Figure S1. Parameter recovery analysis

(A) True vs. recovered parameter values for each of the five free model parameters, across all library entries (n = 344). Each point represents one library entry, coloured by the best-match loss (lighter = lower loss). The dashed line indicates identity; the solid line and shaded band show the linear fit ± 1 residual standard deviation. Pearson r values are shown for each parameter.

(B) Left: Recovery correlation (Pearson r, filled bars) and Spearman r (open circles) for each parameter. Dashed and dotted lines indicate r = 0.70 and r = 0.50 thresholds respectively. Right: Recovery correlation as a function of simulated noise level (expressed as a percentage of the per-metric biological standard deviation), illustrating robustness of well-identified parameters to stochastic variability.

(C) Degeneracy structure of the fitting procedure. Left: Distribution of parameter spread within the near-optimal matching set (entries within 10% of the minimum loss) for each parameter, expressed as a percentage of the total parameter range; lower values indicate higher identifiability. Centre: Recovery errors for k_shelter and k_threat plotted against each other, coloured by best-match loss; correlated errors indicate a trade-off degeneracy between these parameters. Right: Cross-parameter confusion matrix showing Pearson correlations between all pairs of true and recovered parameters; diagonal entries reflect direct recovery and off-diagonal entries reflect cross-parameter confusion.

## 1.2. Model Comparison

### 1.2.1. Reduced Model Variants

Across all three representative mice, the full model achieved lower weighted loss (Fig. S2(A)) than all reduced variants. The loss hierarchy was consistent: full < no D module << no T module ≈ only M module, and this ordering was preserved under Bayesian Information Criterion (BIC), confirming it cannot be attributed to the full model's larger parameter count (Fig. S2(B)).

Per-metric decomposition on the median mouse revealed interpretable and architecturally motivated failure modes. The No D model failed specifically on shelter↔corridor transition frequency, consistent with the prediction that without the Danger Context module the agent over-investigates and fails to execute timely shelter returns. The No T and Only M models produced nearly identical loss profiles, with collapsed spatial entropy and near-zero chamber transitions. This convergence reflects a fundamental architectural failure: in the No T variant, the Danger Context module receives a fixed threat signal in the absence of dynamic identity inference from the Threat Identification module, causing the optimizer to compensate by inverting the shelter preference (the mouse “dislikes” the shelter), a degenerate solution that removes all structured behavior. The near-equivalence of No T and Only M thus demonstrates that the Danger Context module contributes no functional benefit without upstream threat identity inference.

These results demonstrate that each module contributes a specific, non-redundant computational role that cannot be recovered through parameter compensation in reduced variants.

### 1.2.2. Alternative Baseline Models

We further developed 2 alternative models: a Lévy walk model, and a hidden Markov model. The Lévy walk approximated median mouse behavior reasonably well but was systematically worse at both behavioral extremes, compared to the active inference model (Fig. S2(C)). For the "anxious" mouse, the Lévy walk model could not simultaneously reproduce high shelter occupancy (Lévy walk model: 26.5% ± 17.1%, target: 82.4%) and structured shelter↔corridor transitions (Lévy walk model: 2.55 ± 1.20, target: 15). These are behaviors that in the active inference model emerge from periodic resolution of the epistemic-safety conflict by the Danger Context module. For the "curious" mouse, sustained social investigation time was severely underfit (model: 25.3% ± 7.6%, target: 56.0%), accounting for 87% of total loss. This suggests that directional persistence alone cannot generate sustained proximity to a specific arena zone without an explicit epistemic drive. The average mouse was well-approximated, but this should be interpreted cautiously: moderate behavior in a bounded arena is broadly consistent with undirected movement, and the Lévy walk fails to account for chamber transition structure even in this best case. While the Lévy walk achieves comparable or lower BIC (Fig. S2(D)) than the active inference model owing to its minimal parameterization, this should not be interpreted as evidence of equivalent explanatory power. The primary goal of this study is not to find the most parsimonious statistical description of movement, but to construct an interpretable mechanistic account of how adverse social experience reshapes decision-making. The Lévy walk's parameters (i.e, tail exponent, directional persistence, and stationary probability) describe the statistics of movement without providing any account of why an animal moves as it does, and are not mappable onto cognitive or neural quantities.

The hidden Markov Model (HMM) consistently recovered a functionally interpretable partition of the action space into immobile, rightward-mobile, and leftward-mobile states - broadly paralleling dwelling, approach, and retreat - but without spatial grounding. Despite this emergent structure, the model failed systematically across all three phenotypes. For the "anxious" mouse, loss was 35.94 ± 9.77, with shelter occupancy alone accounting for the dominant error ($w \cdot z^2 = 25.1$): the model can generate high immobility but cannot spatially confine it to the shelter zone. For the "curious" mouse, loss was 14.02 ± 5.30, with sustained investigation severely underfit ($w \cdot z^2 = 9.15$), as directed approach toward a specific goal location cannot be generated without an explicit goal representation. The average phenotype was fit most closely (loss: 7.87 ± 2.01), with residual errors concentrated in chamber transition frequency and spatial entropy. Despite having 20 free parameters compared to 5 in the active inference model, the HMM achieved substantially higher loss across all phenotypes, a finding also visible in BIC (Fig. S2(D)).

Together, the weaknesses of both alternative baselines are architectural rather than parametric. That is, the computations required to reproduce extreme behavioral phenotypes are absent from these models by construction and cannot be recovered by parameter tuning. Importantly, neither baseline, whether statistically flexible like the

HMM or parameter-efficient like the Lévy walk, provides the mechanistic description necessary to address the core questions of this study. Goodness of fit is a necessary but not sufficient criterion for a model of behavior.

*Supplementary Figure S2*

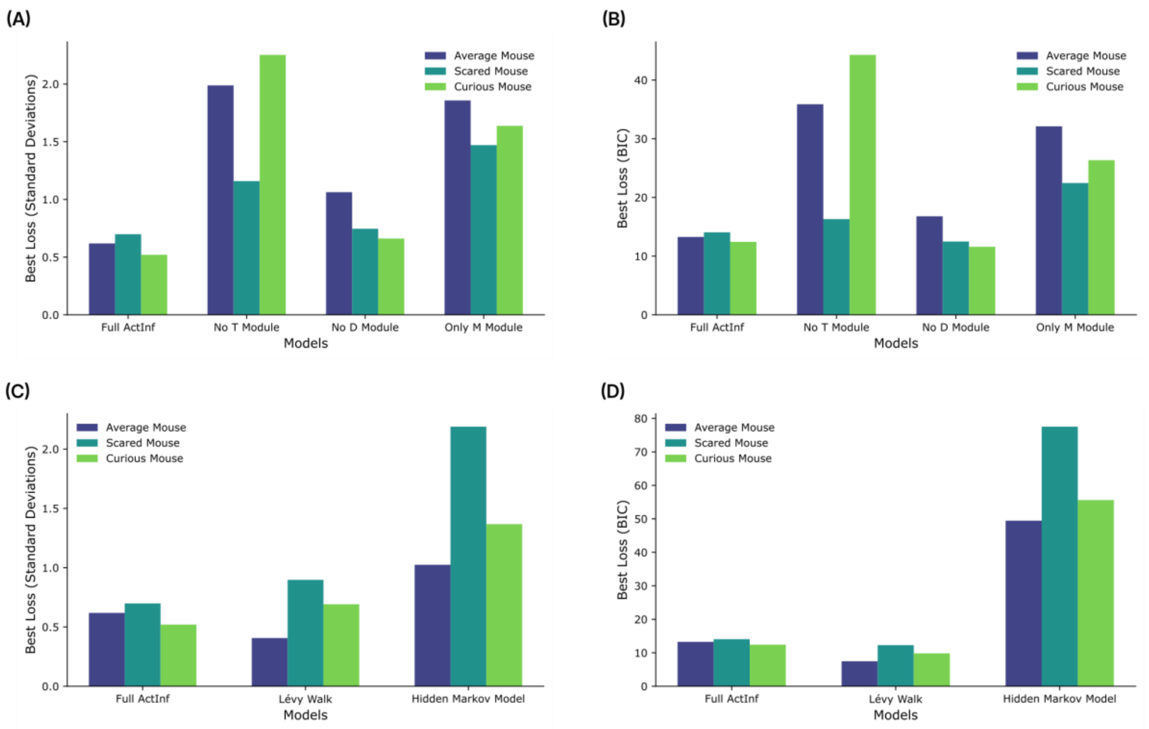


Supplementary Figure S2. Model comparison across reduced and alternative model variants

(A) Fit error for the full model and three reduced variants across three representative mice. Reduced models receive an equivalent optimization budget despite having fewer free parameters, giving them a relative advantage. The full model achieves lower error across all three phenotypes, with no T and M only performing near-equivalently, reflecting the functional dependence of the Danger Context module on upstream threat identity inference.

(B) Bayesian Information Criterion (BIC = loss + k·ln(N), k = number of free parameters, N = 8 metrics) for the same model variants, penalizing parameter count. The full model advantage is preserved under BIC, confirming it cannot be attributed to greater model complexity.

(C) Fit error for the active inference model and two alternative baselines (Lévy walk, HMM) across the same three representative mice. Both baselines systematically underperform at behavioral extremes despite comparable or greater parameter counts, with failures concentrated in phenotype-defining metrics.

(D) BIC for the same alternative model comparison. The HMM shows substantially higher BIC than the active inference model across all phenotypes due to its 20 free parameters, confirming its failures are architectural. The Lévy walk achieves comparable or lower BIC owing to its minimal parameterization ($k = 3$); however, its parameters (tail exponent, directional persistence, and stationary probability) carry no mechanistic interpretation: they describe the statistics of movement without accounting for why an animal moves as it does. In contrast, the active inference model's parameters correspond directly to interpretable cognitive quantities (threat sensitivity, shelter preference, sensory precision), enabling principled hypotheses about the computational and neural mechanisms underlying behavioral variation.

## 1.3. Temporal Split Validation

To assess whether the model generalises beyond the data it was fitted to, we split each mouse's 2000-step trajectory at timestep 1000 and evaluated whether parameters matched to the first half predicted held-out second-half behavior. Before fitting, we checked for non-stationarities. P*re-defeat* sessions did not show systematic and clear non-stationarities: no behavioral metric differed significantly between halves (Wilcoxon signed-rank, all $p > 0.1$, all $|r| < 0.37$; Figure S3(A), Table S1). *Post-defeat* sessions showed one significant within-session effect: shelter→corridor transitions increased in the second half ($p=0.018$, $r=0.63$, consistent across 11/13 mice; Figure S3(A), Table S1), with no corresponding increase in corridor→chamber transitions (non-significant), indicating mice make more shallow exploratory sallies from shelter without approaching the threat further.

After first fitting the model to the first 1000 timesteps, we found that the parameters overall fit the second 1000 timesteps well. The model produced unbiased predictions for the two primary metrics (time in shelter and time investigating) with median absolute errors of 0.6–0.7σ on held-out data, comparable to the original 0.56σ fit quality (Figure S3(B)). Shelter↔corridor transition counts were systematically underestimated in the second half of both sessions (Wilcoxon one-sample test, Benjamini-Hochberg correction across 8 metrics: pre-defeat $p=0.035$, post-defeat $p=0.002$), potentially indicating the model does not fully capture very slow learning inference dynamics at the timescale of an entire session. Corridor↔chamber transitions were not significantly mispredicted in post-defeat sessions, though a minor over-prediction was observed pre-defeat (FDR $p=0.041$–$0.045$), suggesting the model

produces slightly more chamber exploration than observed in the first session. The systematic underestimation of shelter exits is the dominant and consistent pattern across both sessions. The Danger Context belief decay parameter ($\eta_D$), held fixed in the current implementation, is a natural candidate for capturing this within-session dynamic in future work.

*Supplementary Figure S3*

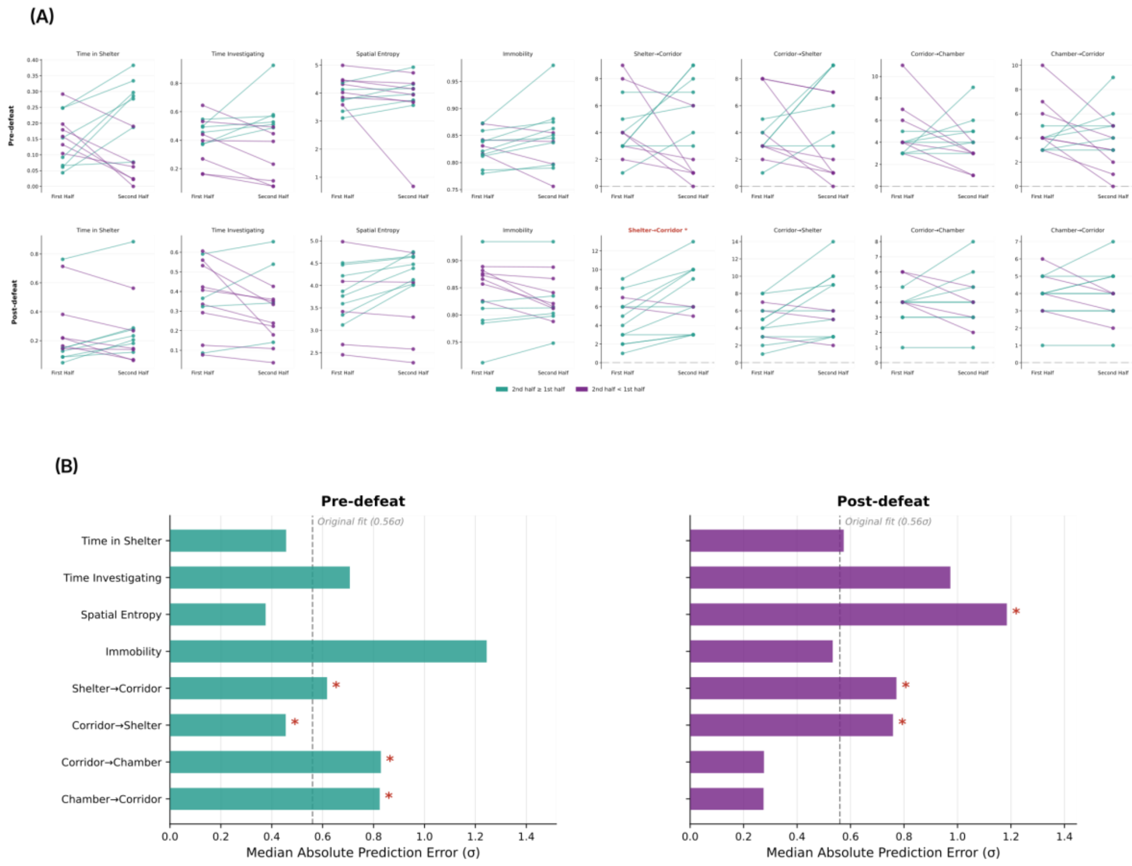


Supplementary Figure S3. Within-session stationarity and out-of-sample generalization of the model

(A) Pre-defeat sessions are stationary: no behavioral metric differs between first and second halves (Wilcoxon signed-rank, all $p > 0.1$, all $|r| < 0.37$). Post-defeat, only shelter→corridor transitions increase in the second half ($p = 0.018$, $r = 0.63$; 11/13 mice), with no change in corridor→chamber transitions, indicating more shallow excursions without deeper approach.

(B) Parameters fit to the first half generalize to held-out second-half data. Primary metrics (time in shelter, time investigating) are predicted without bias (median absolute error 0.6–0.7σ; cf. 0.56σ in-sample). Shelter↔corridor transitions are underestimated in both sessions (BH-corrected Wilcoxon one-sample: pre $p = 0.035$, post $p = 0.002$), whereas corridor↔chamber transitions are not mispredicted, indicating a selective failure to capture increased shelter-exit behavior.

*Table S1*

| Metric | Pre-defeat Median \|Error\| (σ) | Post-defeat Median \|Error\| (σ) | Pre-defeat FDR p | Post-defeat FDR p |
|---|---|---|---|---|
| Time in Shelter | 0.46 | 0.57 | 0.622 | 0.346 |
| Time Investigating | 0.71 | 0.97 | 0.145 | 0.946 |
| Shelter→Corridor | 0.62 | 0.77 | 0.035* | 0.002* |
| Corridor→Shelter | 0.46 | 0.76 | 0.035* | 0.002* |
| Corridor→Chamber | 0.83 | 0.28 | 0.045* | 0.946 |
| Chamber→Corridor | 0.82 | 0.27 | 0.041* | 0.946 |
| Spatial Entropy | 0.38 | 1.18 | 0.091 | 0.012* |
| Immobility | 1.24 | 0.53 | 0.091 | 0.345 |

Table S1. Temporal split prediction errors by metric. Median absolute prediction error (in units of population standard deviation, σ) for each behavioral metric, computed by matching model parameters to the first 1000 steps of each session and evaluating predictions on the held-out second 1000 steps. Note: p-values here refer to whether model prediction errors are significantly different from zero (i.e. whether the model systematically over- or under-predicts each metric), and are distinct from the stationarity tests reported, which compare empirical first-half vs second-half behavioral metrics directly. P-values are from one-sample Wilcoxon signed-rank tests of normalized prediction errors against zero, Benjamini-Hochberg corrected across 8 metrics within each session. Asterisks indicate FDR-corrected $p < 0.05$. Original cohort, n=13 mice.

## 1.4. Generalization to Independent Dataset

To test generalization to entirely new animals, we recorded six additional mice (3 control, 3 defeat) using the same experimental protocol. Without any re- simulation, we identified the best-matching parameter set from the existing library for each new mouse using the same loss function. The library generalized well: median fit error was 0.53σ, essentially identical to the original cohort benchmark of 0.56σ, with 11/12 mouse × phase combinations fit within 1σ (Figure S4, Table S2). This demonstrates that a library constructed from a small number of representative individuals spans a behavioral manifold sufficient to describe new animals without re-optimization.

*Supplementary Figure S4*

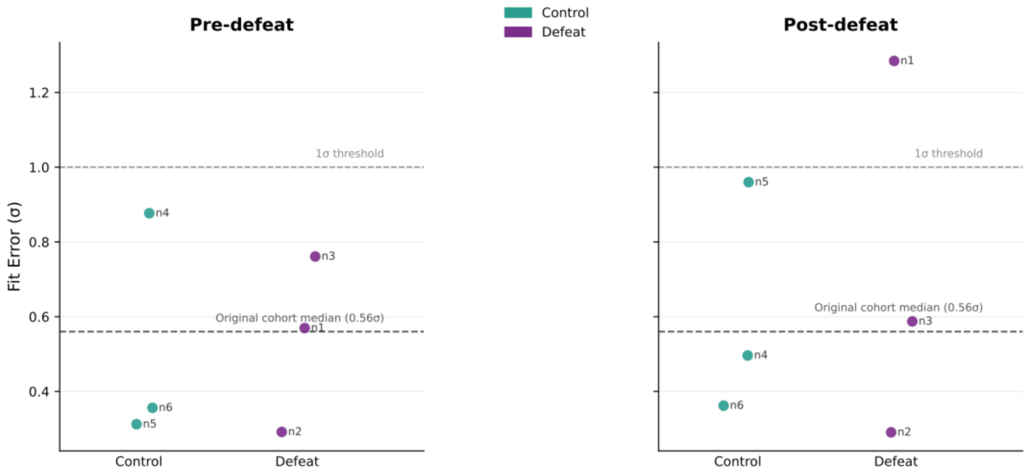


Supplementary Figure S4. Generalization to novel animals using a fixed parameter library

Model performance on six previously unseen mice (3 control, 3 defeat), evaluated without re-fitting by selecting the best-matching parameter set from a precomputed library. Points show per-mouse fit error (σ units) for pre-defeat (left) and post-defeat (right) sessions. Dashed lines indicate the original cohort median (0.56σ) and 1σ threshold. Median error across new animals is 0.53σ, with 11/12 mouse × phase combinations within 1σ, demonstrating that a library derived from a small set of representative individuals generalizes to new subjects.

*Table S2*

| Mouse ID | Group | Phase | Fit Error (σ) |
|---|---|---|---|
| n1 | Defeat | Pre-defeat | 0.57 |
| n1 | Defeat | Post-defeat | 1.28 |
| n2 | Defeat | Pre-defeat | 0.29 |
| n2 | Defeat | Post-defeat | 0.29 |
| n3 | Defeat | Pre-defeat | 0.76 |
| n3 | Defeat | Post-defeat | 0.59 |
| n4 | Control | Pre-defeat | 0.88 |
| n4 | Control | Post-defeat | 0.5 |

| | | | |
|---|---|---|---|
| n5 | Control | Pre-defeat | 0.31 |
| n5 | Control | Post-defeat | 0.96 |
| n6 | Control | Pre-defeat | 0.36 |
| n6 | Control | Post-defeat | 0.36 |
| Overall median | | | 0.53 |
| Control median | | | 0.43 |
| Defeat median | | | 0.58 |
| Pre-defeat median | | | 0.46 |
| Post-defeat median | | | 0.54 |

Table S2. Parameter library fit quality for independent dataset. Fit error (σ) for each mouse × phase combination in the independent validation dataset (n=6 mice, 3 control, 3 defeat), computed by matching each mouse's full-session behavioral metrics to the existing parameter library without re-optimization or new simulations. $\sigma = \sqrt{(\text{loss}/\Sigma\text{weights})}$. Summary statistics are shown by group and phase. The original cohort benchmark of 0.56σ is provided for reference.

## 1.5. Individual Differences - Susceptible vs Resilient

As an exploratory analysis enabled by the expanded dataset, we asked whether the model's parameter space distinguishes susceptible from resilient individuals within the defeat group. Susceptibility labels were derived empirically for the 6 original cohort defeat mice from a composite behavioral change score (Δt_shelter - Δt_investigating, post- minus pre-defeat), yielding a clean median split: susceptible mice (m24, m26, m14; scores +0.989, +0.871, +0.275) and resilient mice (m17, m23, m16; scores +0.093, +0.081, −0.714).

Post-defeat parameter space showed clean separation on four dimensions, with threat aversion (k_threat) as the dominant axis (Cohen's d=8.16, no overlap: susceptible 1.20–1.38 vs resilient 0.41–0.57; Table S3, Figure S5(A)). This separation is also visible in the post-defeat PCA across all 19 mice (Figure S5(B)). Pre-defeat parameters showed no separation (all $|r| \leq 0.56$), indicating the distinction reflects defeat-induced reorganization rather than pre-existing individual differences. A threshold classifier on k_threat alone (threshold = 0.883, midpoint between groups) correctly classified all 6

original cohort defeat mice, and applied prospectively to the 3 new cohort defeat mice, correctly classified all 3 (Table S4).

Analysis of per-mouse Trauma Vectors (post- minus pre-defeat parameter shifts) revealed that susceptible and resilient mice shift in the same computational direction after defeat (cosine similarity = +0.932), differing primarily in magnitude (||susceptible|| = 3.20 vs ||resilient|| = 0.25; Figure S5(C)). Both groups oppose the control direction (cosine ≈ −0.98). This suggests susceptibility reflects a quantitatively larger reorganization along shared computational axes rather than a qualitatively distinct response to defeat.

*Supplementary Figure S5*

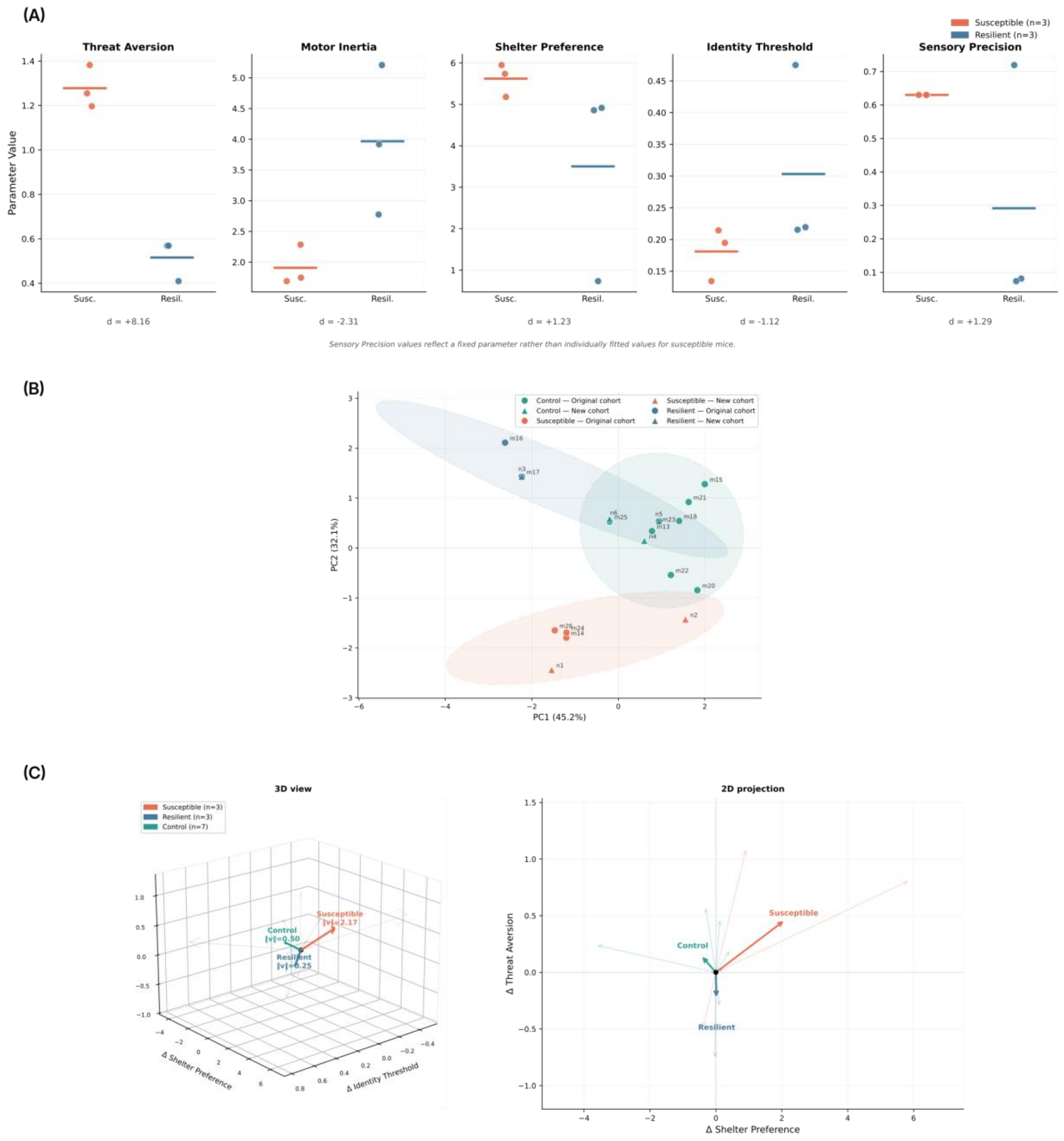


Supplementary Figure S5. Model parameter space separates susceptible and resilient mice post-defeat

(A) Post-defeat parameter values by phenotype group. Strip plots showing individual parameter values for susceptible (orange, n=3) and resilient (steel blue, n=3) mice in the post-defeat session, for each of the five model parameters ordered by effect size (left to right). Horizontal bars indicate group means. Cohen's d values are shown below each panel title. Sensory Precision values for susceptible mice reflect a fixed parameter rather than individually fitted values.

(B) PCA of post-defeat parameters across all mice. Scatter plot of the first two principal components (PC1 45.2%, PC2 32.1%) of the five post-defeat model

parameters, standardized across all 19 mice. Points are colored by phenotype group (Control = teal, Susceptible = orange, Resilient = steel blue) and shaped by cohort (original cohort = circle, new cohort = triangle). 95% confidence ellipses are shown for each group. Mouse IDs are indicated. Parameters were standardized to zero mean and unit variance prior to PCA.

(C) Defeat-induced parameter shifts by phenotype group. Left: 3D arrow plot showing mean trauma vectors (post- minus pre-defeat parameter shifts) for susceptible (orange), resilient (steel blue), and control (teal) groups, with individual mouse vectors shown as faded arrows. Right: 2D projection onto Δ Shelter Preference (x-axis) and Δ Threat Aversion (y-axis). Arrow length is proportional to vector magnitude (||susceptible|| = 3.20, ||resilient|| = 0.25, ||control|| = 0.50). Individual mouse vectors are shown as faded arrows in the corresponding group color. Original cohort mice only (n=13).

*Table S3*

| Parameter | Susceptible Mean (SD) | Resilient Mean (SD) | Cohen's d |
|---|---|---|---|
| Threat Aversion (k_threat) | 1.278 (0.095) | 0.516 (0.092) | 8.16 |
| Motor Inertia (bias_stay) | 1.908 (0.328) | 3.967 (1.217) | -2.31 |
| Shelter Preference (k_shelter) | 5.621 (0.398) | 3.503 (2.397) | 1.23 |
| Identity Threshold (id_threshold) | 0.181 (0.042) | 0.303 (0.149) | -1.12 |
| Sensory Precision (sensory_slope) | 0.630 (0.000) | 0.291 (0.291) | 0.33 |

Table S3. Post-defeat parameter effect sizes between susceptible and resilient mice. Group means, standard deviations, and Cohen's d for each model parameter, comparing susceptible (n=3: m14, m24, m26) and resilient (n=3: m16, m17, m23) mice in the post-defeat session. Parameters are sorted by |Cohen's d| in descending order. Cohen's d was computed using pooled standard deviation. Susceptible Sensory Precision values reflect a fixed parameter rather than individually fitted values. Parameter assignments used the unique assignment procedure described in Methods.

*Table S4*

| Mouse | Post-defeat k_threat | Predicted Label | Empirical Score | Empirical Label | Correct |
|---|---|---|---|---|---|
| n1 | 1.477 | Susceptible | 0.463 | Susceptible | Yes |

| | | | | | |
|---|---|---|---|---|---|
| n2 | 1.156 | Susceptible | 0.38 | Susceptible | Yes |
| n3 | 0.569 | Resilient | -0.002 | Resilient | Yes |

Table S4. Classification of new cohort defeat mice. For each new cohort defeat mouse (n=3), the post-defeat Threat Aversion parameter (k_threat) from the best-matching library entry is compared against the classifier threshold of 0.883 (midpoint between the highest resilient value of 0.569 and lowest susceptible value of 1.197 in the original cohort) to generate a predicted susceptibility label. Empirical susceptibility scores (Δt_shelter − Δt_investigating) and labels are derived independently from behavioral metrics.

## 1.6. Single-timestep calculation example in the Threat Identification module

To illustrate the mechanics of belief updating and action selection in the discrete active inference framework, we trace a single perception–action cycle through a simplified version of the Threat Identification (T) module. Although the full model operates over $|S| = 20$ identity–proximity states and $|O| = 10$ observation levels, the core computations are identical in structure to the 2-state, 3-observation case presented here.

### 1.6.1. Perception step

The T module maintains a belief vector $q(s) \in \mathbb{R}^2$ over hidden identity states {Not Threat, Threat}, initialized to a uniform prior $q(s) = [0.5, 0.5]^T$, reflecting maximal uncertainty about the conspecific's identity at the start of an episode. The likelihood matrix $A \in \mathbb{R}^{(3\times2)}$ encodes how each identity state generates sensory observations of conspecific signal intensity {low, medium, high}:

A = [ 0.8   0.1

0.15  0.3

0.05  0.6 ]

Column 1 (Not Threat) assigns high probability to weak signals; column 2 (Threat) assigns high probability to strong signals, consistent with the distance-dependent sensory precision governed by the sensory_slope parameter (Methods Section 5.6.2). Upon receiving observation o = "high" (consistent with close proximity to a conspecific emitting a strong olfactory signal) the perception step executes an

elementwise product between the corresponding likelihood row and the prior belief, followed by normalization:

$q(s \mid o) \propto A[2, :] \odot q(s) = [0.05, 0.6] \odot [0.5, 0.5] = [0.025, 0.3] \rightarrow q(s \mid o) = [0.077, 0.923]$

The agent's belief shifts from maximal uncertainty to 92.3% confidence that the conspecific is a Threat. As this exceeds the identity confidence threshold (Methods Section 5.4), this belief is propagated immediately to the Danger Context (D) module, bypassing the D module's regular update ticker and triggering the transition from epistemic to pragmatic drive described in Section 3.2.

### 1.6.2. Action selection step

The T module evaluates two candidate policies: {Null, Approach}. Policy selection proceeds by computing the Expected Free Energy G for each candidate. Epistemic value - the primary driver of T module action - is computed as the expected reduction in belief entropy under each policy:

$EV(\pi) = H[q(o)] - q(s)^T \cdot H[A]$

where $H[q(o)] = -\Sigma_o\, q(o) \ln q(o)$ is the entropy of predicted observations and $q(s)^T \cdot H[A]$ is the expected entropy of the likelihood under the current belief, capturing residual ambiguity in the observation model itself. With belief now concentrated at $q(s|o) \approx [0.08, 0.92]$, the entropy $H[q(s|o)]$ is low ($\approx$ 0.36 nats), and further approach yields negligible additional information gain. Consequently, the epistemic value of the Approach policy is near zero, and the Null policy is selected. Control over the Motor (M) module's preference vector C_M reverts to default shelter-seeking, and the Danger Context module's Avoid action amplifies this preference, producing the ballistic flight trajectory shown in Figure 3.

This example demonstrates how a single high-precision observation is sufficient to collapse uncertainty, trigger inter-module communication, and redirect behavior - without requiring any explicit rule-based switching. The same matrix operations, scaled to the full state and observation spaces, govern all three modules at every timestep of the simulation (Algorithm 1).

# 2. Methods

## 2.1. Parameter Recovery

To assess whether the fitting procedure reliably identifies the parameters underlying observed behavior, we performed a parameter recovery analysis on the optimization library (n = 344; see Section 5.6.4). For each library entry, its simulated behavioral metrics were treated as a synthetic target, and the best-matching entry among the remaining library entries was identified by minimizing the same weighted z-score loss function used in the original fitting procedure (Section 5.6.3). Metric normalisation used standard deviations derived from the experimental cohort (n = 13 mice) rather than from the library itself, to ensure the loss function reflected biologically meaningful variance. Recovery quality was quantified as the Pearson correlation between true and recovered parameter values across all library entries.

To assess robustness to simulation stochasticity, we repeated the analysis across a range of additive noise levels (0–100% of the biological standard deviation per metric), computing recovery correlations at each level. Library metrics represent averages over three independent simulation runs per parameter configuration; accordingly, simulation noise enters the effective normalisation as

$\sigma_{eff} = \sqrt{(\sigma_{bio}^2 + \sigma_{noise}^2)}$

where $\sigma_{noise}$ is the per-metric within-configuration standard deviation estimated from repeated runs.

## 2.2. Model Comparison

### 2.2.1. Reduced Model Variants

To assess the necessity of each architectural component, we compared the full heterarchical model against three reduced variants obtained by systematically ablating individual modules: a model without the Danger Context module (noD), a model without the Threat Identification module (noT), and a motor-only model (M_only) retaining only the Spatio-Motor module. Each variant was fit to three representative mice spanning the behavioral spectrum (scared/anxious, median, curious) using the identical Bayesian optimization pipeline as the full model (Tree-Structured Parzen Estimator, 100 trials, identical loss function and metric definitions). Critically, reduced variants have fewer free parameters (noD: 4, noT and M_only: 3) than the full model (5), yet receive the same optimization budget. This gives reduced models a relative advantage in trials-per-parameter. To account for model complexity, we report both raw weighted loss and Bayesian Information Criterion (BIC = loss + k·ln(N), where k is the number of free parameters and N = 8 metrics that compose the loss), treating the

loss as proportional to $-2 \ln \hat{L}$ (since it is the sum of squared z-scores) under an assumption of independent Gaussian errors across metrics.

### 2.2.2. Lévy Walk Baseline

We implemented a Lévy walk as a parameter-matched statistical baseline. The agent receives no sensory input and has no zone preferences, governed by three parameters: μ (Lévy tail exponent), κ (von Mises directional persistence), and p_stay (stationary probability). Step lengths are drawn from $P(l) \propto l^{(-\mu)}$ over {1,...,50}; turning direction is sampled from a von Mises-weighted categorical distribution, reducing to uniform at $\kappa = 0$. The agent operates on the identical 57-state arena with identical boundary conditions. Parameters were fit independently for each representative mouse using the identical optimization pipeline and trial budget as the active inference model (100 trials), ensuring a controlled comparison. Best-fit metrics were estimated by averaging across 20 independent simulations at optimal parameters.

### 2.2.3. Hidden Markov Model Baseline

We implemented a 3-state multinomial Hidden Markov Model (HMM) as a descriptive baseline capturing statistical sequence structure without positing internal beliefs, goals, or planning. The HMM comprises 3 hidden states with multinomial emissions over 5 discrete actions, yielding 20 free parameters. Empirical trajectories were converted to action sequences from consecutive grid state transitions; geometrically impossible transitions (diagonal jumps: 65.8%, horizontal 2-cell jumps: 25.9%, together 91.7% of anomalies, 2.54% of total) were resolved by intermediate state insertion, with remaining anomalies (0.2%) handled by sequence splitting. One HMM was fit per mouse per session using Baum-Welch (200 iterations, tolerance $1\times10^{-4}$, 30 random restarts) with Dirichlet-initialized parameters (transitions: $\alpha = 1$, emissions: $\alpha = 0.5$). Loss was evaluated identically to the active inference model across the same three representative mice; BIC was computed as $-2 \ln \hat{L} + 20 \ln N$.

## 2.3. Temporal Split Validation

Full-session 2000-step trajectories were split at timestep 1000. All 8 behavioral metrics were computed independently on each half using the identical pipeline as the main analysis. For parameter matching, transition count targets were scaled by 0.5 to account for trajectory length, since these metrics accumulate over time; fractional metrics and entropy were used unchanged, as these deviate less than 0.5σ between full-session and first-half values (transition counts deviate 0.7–1.1σ due to scaling alone). The best-matching library entry for each mouse's first half was identified using the same loss function as the main analysis. Second-half predictions were obtained from timesteps 1001–2000 of the matched simulation history, naturally carrying forward belief states from the first half without reinitialization.

## 2.4. Generalization to Independent Dataset

Six new mice were recorded using the identical experimental protocol. DLC tracking data were processed with the same pipeline. All 12 files loaded successfully with 96–99% confidence-filtered rows retained. Full-session behavioral metrics were computed for each mouse × phase. For each combination, the best-matching library entry was identified using the same loss function as the main analysis, with each mouse's own metrics and unscaled transition counts (full 2000-step metrics on both sides). No parameter optimization or new simulations were performed.

## 2.5. Individual Differences - Susceptible vs Resilient

Susceptibility scores were computed as $\Delta t\_shelter - \Delta t\_investigating$ and a median split applied. Post-defeat parameter sets were obtained using a unique assignment procedure solving the assignment problem across all original cohort mice - minimizing total cohort loss subject to no two mice sharing a library entry - independently for pre- and post-defeat sessions. Effect sizes between subgroups were quantified using Cohen's d and rank-biserial r (Mann-Whitney U). Trauma vectors were computed as the difference between post- and pre-defeat matched parameter sets, restricted to three parameters (id_threshold, k_shelter, k_threat) consistent with the main manuscript analysis. The k_threat threshold classifier used the midpoint between the highest resilient value (0.569) and lowest susceptible value (1.197).
